# Full-Thickness Regeneration of the Hard Palate Using Bioengineered Mucoperiosteal Scaffolds in a Porcine Model

**DOI:** 10.3390/biomimetics11090652

**Published:** 2026-09-10

**Authors:** Maria Ida Rizzo, Maria Emiliana Caristo, Chiara Ribaldone, Simone Faustino Maria Marino, Giorgio Spuntarelli, Anna Chiara Contini, Luigi Dall’Oglio, Luigi Tomao, Mattia Algeri, Stefano Tedesco, Gianantonio Pozzato, Cristiano De Stefanis, Antonello Cardoni, Lorenzo Lupoi, Camilla Codazzi, Lucia Leone, Mario Zama, Massimiliano Raponi

**Affiliations:** 1 Department of Plastic and Maxillofacial Surgery, Bambino Gesù Children’s Hospital, IRCCS, 00165 Rome, Italy; mariaida.rizzo@opbg.net (M.I.R.); simone.marino@opbg.net (S.F.M.M.); mario.zama@opbg.net (M.Z.); 2 Centro Ricerche Sperimentali, Università Cattolica del Sacro Cuore, 00168 Rome, Italy; mariaemiliana.caristo@unicatt.it (M.E.C.); lorenzo.lupoi@unicatt.it (L.L.); 3 Gastroenterology and Nutrition Unit, Digestive Endoscopy and Surgery, Bambino Gesù Children’s Hospital, IRCCS, 00165 Rome, Italy; annachiara.contini@opbg.net (A.C.C.); luigi.dalloglio@opbg.net (L.D.); 4 Department of Pediatric OncoHematology and Cell and Gene Therapy, Bambino Gesù Children’s Hospital, IRCCS, 00165 Rome, Italy; luigi.tomao@opbg.net (L.T.); mattia.algeri@opbg.net (M.A.); 5 Telea Biotech and Telea Electronic Engineering, 36066 Sandrigo, Italy; stefano.tedesco@teleamedical.com (S.T.); gianantonio.pozzato@teleamedical.com (G.P.); 6 Research Laboratories, Histology Core Facility, Bambino Gesù Children’s Hospital, IRCCS, 00165 Rome, Italy; cristiano.destefanis@opbg.net; 7 Department of Anatomical Pathology, Bambino Gesù Children’s Hospital, IRCCS, 00165 Rome, Italy; antonello.cardoni@opbg.net; 8 Department of Neuroscience, Università Cattolica del Sacro Cuore, 00168 Rome, Italy; camilla.codazzi@unicatt.it (C.C.); lucia.leone@unicatt.it (L.L.); 9 Fondazione Policlinico Universitario A. Gemelli, IRCCS, 00168 Rome, Italy; 10 Management and Diagnostic Innovations & Clinical Pathways Research Area, Medical Directorate, Bambino Gesù Children’s Hospital, IRCCS, 00165 Rome, Italy

**Keywords:** cleft palate, mesenchymal stem cells, tissue engineering, decellularized mucoperiosteum, bone regeneration, bioengineered scaffold

## Abstract

Cleft palate is a congenital anomaly that causes functional and esthetic challenges, and the hard palate is essential for feeding, speech, and separation of the oral and nasal cavities. Yet, current reconstructive techniques do not restore its bony component. Building on previous in vitro work showing that decellularized palatal mucoperiosteum, microperforated with Quantum Molecular Resonance (QMR^®^) technology and recellularized with mesenchymal stem cells, preserves the collagen microenvironment, supports engraftment, and shows osteoinductive potential, this study evaluated the early feasibility and regenerative potential of bioengineered mucoperiosteal scaffolds (BEMS) in Landrace pigs model. Bone marrow was collected from recipient pigs to isolate pBM-MSCs. Donor palatal mucoperiosteum was decellularized, microperforated, and recellularized with these cells to generate BEMS. After surgical creation of a cleft palate, four pigs received BEMS, and two controls underwent standard palatoplasty. At one month, scaffold-treated animals showed early mucosal and osseous regeneration, including neo-epithelium, connective tissue, and new bone formation, without clinical or routine histological signs of acute rejection. SPARC (Secreted protein acidic and rich in cysteine) expression supported osteogenic activity. Regenerated palates were stable and fracture-resistant, whereas controls showed incomplete repair and fractures. These findings suggest that BEMS may address limitations of conventional palatal reconstruction and support further investigation for human palatal bone regeneration.

## 1. Introduction

Cleft lip and palate (CL/P) is one common congenital craniofacial malformation, affecting approximately 1 in 700 live births [1]. These anomalies cause significant morphological and functional impairments, including difficulties in feeding, speech, and hearing, as well as psychosocial challenges. Despite the availability of various surgical protocols, achieving complete and stable restoration of hard palate bone continuity remains an unresolved challenge. Current techniques primarily reconstruct soft tissues, often leaving residual bony defects that can result in oronasal fistulas with nasal regurgitation of food, impaired maxillary growth, and limitations to future orthognathic procedures precluding Le Fort I osteotomy if the patient needs to advance the maxilla [2,3].

Autologous bone grafting from the iliac crest is the standard treatment for alveolar clefts and has shown reliable clinical outcomes. However, this approach is limited to alveolar repair, does not address hard-palate defects, and cannot be performed in the neonatal period. Early palatal repair (within the first 6–8 months of life) is essential to support normal development.

These limitations have driven interest in regenerative medicine and tissue engineering as alternative strategies for bone regeneration in CL/P patients. Recent advances focus on developing biocompatible scaffolds capable of supporting osteogenic differentiation and integration with host tissues. Synthetic materials such as beta-tricalcium phosphate and hydroxyapatite offer osteoconductive properties but face limitations in mechanical performance and tissue integration. Conversely, natural biomaterials, particularly decellularized extracellular matrices, have emerged as promising scaffolds due to their ability to closely replicate native tissue architecture and promote cellular infiltration and vascularization [4,5,6].

Previous studies have demonstrated the potential of palatal-derived biomaterials and decellularized extracellular matrix for tissue engineering [7]. More recent biomaterial-based strategies have further explored approaches for palatal wound healing, although most have primarily focused on soft-tissue repair rather than complete regeneration of the hard palate [8]. In our previous in vitro study, we developed a decellularized porcine mucoperiosteal scaffold modified by QMR-assisted microperforation and demonstrated its ability to support MSC adhesion, proliferation, and osteogenic differentiation [9]. However, whether this engineered scaffold could promote full-thickness regeneration of the hard palate in a large-animal model remained unexplored. The present study was therefore designed to address this gap.

Building on these findings, the present study investigates the in vivo feasibility of BEMS transplantation in a porcine model of iatrogenic cleft palate. This study aims to evaluate the engraftment and early bone formation potential in a pilot preclinical study of the bioengineered decellularized palatal mucoperiosteum scaffold recellularized with recipient BM-MSCs and to evaluate its engraftment and early bone formation one month after transplantation.

Our primary objective was to evaluate the feasibility and early regenerative response of the scaffold in hard palate reconstruction while assessing its in vivo tolerance post-transplantation. To reach this goal, we harvested palatal mucoperiosteum from donor pigs and subjected them to decellularization protocols leaving only the extracellular matrix (ECM). Bone marrows from recipient pigs were then sampled to isolate and selectively expand autologous BM-MSCs, which were then used in the ECM recellularization phase. The cohort of recipient pigs, previously surgically treated to induce an iatrogenic cleft palate, served as the experimental model for engineered mucoperiosteum transplantation. One month after implantation, macroscopic and microscopic assessment provided preliminary evidence of scaffold integration and early regenerative activity, supporting further investigation of this bioengineering approach for CL/P repair.

## 2. Materials and Methods

The study was conducted in accordance with ethical standards and principles expressed in the Declaration of Helsinki, approved by the Scientific Committee of the Bambino Gesù Children’s Hospital, and supported by the Italian Ministry of Health through Current Research funds. All procedures involving animals complied with the guidelines set by the body responsible for animal welfare (OPBA) and adhered to current Italian (Legislative Decree no. 26/2014) and European (Directive 2010/63/EU) legislation. The experimental protocol, entitled “Genetic Reprogramming of the Mucoperiosteum for Transplantation and Bone Regeneration”, was approved by the Italian Ministry of Health (Authorization No. 91/2022-PR, issued on 7 February 2022). All pigs used in the study were supplied by Azienda Agricola Pace Maria Antonietta, Rome, Italy (supplier code: 091RM801), and were housed and handled at the Experimental Research centre of the Catholic University of the Sacred Heart, Rome, Italy, authorized by the Italian Ministry of Health (authorization no. 29/2025-UT, issued on 10 October 2025).

In vivo experiments: 8 pigs Landrace breed were enrolled: 2 donors; 4 recipients; 2 controls.

Adult donor pigs were selected in order to simulate adult human donors. Only two adult pigs were used as donors, from which four mucoperiosteal scaffolds could be generated. This approach was deliberately chosen to adhere to the principles of animal refinement, minimizing the number of animals while still obtaining the required experimental tissues.

The sample size was selected in accordance with ethical principles of animal reduction and the exploratory nature of the study.

Recipient and control pigs were selected at 4–6 months of age to simulate infants affected by CL/P.

The mucoperiosteum was harvested from the hard palate of donor pigs and subjected to decellularization protocol, microperforation based on QMR^®^ technology, and recellularization with bone marrow-derived mesenchymal stem cells (MSCs) obtained from recipient pigs, as described below.

All recipient and control pigs underwent surgical induction of an iatrogenic cleft palate.

Controls received traditional palate reconstruction according to the Langenbeck procedure, while recipients received transplantation of the bioengineered mucoperiosteum scaffolds (BEMS).

In the same surgical session, all pigs underwent gastrostomy to ensure adequate nutritional support throughout the experimental period.

### 2.1. Anesthesia, Perioperative Management, and Euthanasia

All surgical procedures were performed under general anesthesia according to standardized veterinary protocols. Donor, recipient, and control pigs underwent pharmacological premedication and intravenous induction followed by orotracheal intubation and maintenance with inhaled isoflurane. Neuromuscular blockade and intraoperative analgesia were administered as appropriate, and physiological parameters were continuously monitored by the veterinary anesthesia team.

Donor animals were euthanized immediately after mucoperiosteum harvesting while still under general anesthesia. Recipient and control pigs were maintained for a one-month postoperative period before euthanasia.

For euthanasia, animals were placed under deep general anesthesia with adequate analgesia, and Tanax was administered intravenously at a dose of 3 mL/10 kg body weight, in accordance with institutional and national guidelines for the care and use of laboratory animals.

### 2.2. Harvesting BM-MSCs from Recipient Pigs and Palatine Mucoperiosteum Harvesting from Donor Pigs

Under general anesthesia, recipient pigs underwent bone marrow aspiration for MSC isolation, while donor pigs underwent harvesting of the mucoperiosteum from the hard palate without palatine artery. Each harvested palatal mucoperiosteum was sagittally split to generate two scaffolds per donor, resulting in a total of four mucoperiosteal scaffolds of 6 × 1.5 cm. These were collected on dry ice in Styrofoam containers, transported to the laboratory within 1 h of collection, and dry-stored at −80 °C. They were then transferred to Telea Biotech laboratory (Vicenza, Italy) [9].

This strategy was implemented in the effort to respect the principles of animal refinement by minimizing donor numbers while providing sufficient scaffolds for the study.

### 2.3. Palate Decellularization

In Telea Biotech, each palate was washed in ultrapure water containing 2% Penicillin–Streptomycin (P/S, Sigma-Aldrich, St. Louis, MO, USA) for 48 h at 4 °C under static conditions and incubated for five repeated cycles at room temperature in 4% sodium deoxycholate (BioXtra ≥ 98.0—Sigma-Aldrich).

Samples were then treated with 2000 Kunitz units (KUs) of DNase-I (Worthington Biochemical Corporation, Lakewood, NJ, USA) in 1 M NaCl for 3 h at 37 °C, subsequently incubated five times under the same conditions, and finally rinsed in ultrapure water containing 2% P/S.

In order to remove decellularization reagents, samples were washed in increasing concentrations of denatured ethanol (ACS Reagent ≥ 99.8%, without additive—Honeywell) and rehydrated overnight in ultrapure water. Scaffolds were then stored in phosphate-buffered saline (PBS, Sigma-Aldrich) containing 1% P/S at 4 °C.

The effectiveness of this decellularization protocol was previously demonstrated, with residual dsDNA levels consistently below 50 ng/mg and preservation of the native collagen-rich extracellular matrix architecture [9].

### 2.4. Scaffold Microperforation

After decellularization, each sample underwent a patented microscopic perforative treatment (patents EP2164536B1, EP2493522B1, EP3870246A1 property of Telea Biotech Srl, Sandrigo, Vicenza, Italy) using a dedicated current generator, which employs Quantum Molecular Resonance (QMR^®^) technology (property of Telea Medical Srl).

The generator was connected to a 300 μm-diameter needle mounted on a 3-axis Cartesian robot (Yamaha model RCX240, Yamaha Motor Co., Ltd., Iwata, Shizuoka, Japan) handpiece. The procedures were conducted in a class-II biosafety cabinet under aseptic conditions.

This treatment created micropores in biological tissues with adjustable dimensions (from tens to hundred microns) and densities up to 1000 pores/cm^2^ without thermal damage or alteration of the surrounding extracellular matrix (ECM) structure [9]. The same QMR^®^-based microperforation approach has previously been applied to decellularized esophageal scaffolds, including in a porcine esophageal substitution model, demonstrating its suitability for the preparation of biological scaffolds intended for tissue engineering and regenerative applications [10,11].

### 2.5. Recellularization in Cell Factory

MSCs were isolated, cultured, and characterized as previously described. Briefly, mononuclear cells were isolated from the bone marrow of four recipient pigs. Cultures were maintained at 37 °C in a humidified atmosphere containing 5% CO_2_. Then, 48 h after seeding, non-adherent cells were removed. Fresh culture medium (DMEM; Euro clone, Pero (Milano), Italy) was replaced twice a week. MSCs were harvested at ≥80% confluence and propagated at 4 × 10^3^ cells/cm^2^. Osteogenic and adipogenic differentiation of MSCs were evaluated based on calcium and fat droplet deposition using Alizarin Red and Oil Red O (Sigma-Aldrich, St. Louis, MO, USA) staining, respectively [9].

Before seeding, a portion of each scaffold was excised. MSCs were harvested, counted, and resuspended at a concentration of 1 × 10^6^ cells/mL. Cells were then seeded onto each decellularized scaffold and immobilized on the bottom of a Petri dish, previously washed and equilibrated in medium.

After 2 h, a sufficient volume of medium was added to cover the scaffolds. Cultures were maintained for 14 days, with three reseeding procedures performed as previously described. A portion of each scaffold was stored at the end of the culture period (day 14) for further testing.

### 2.6. BEMS Transplantation in Recipient Pigs

**Figure 1:**
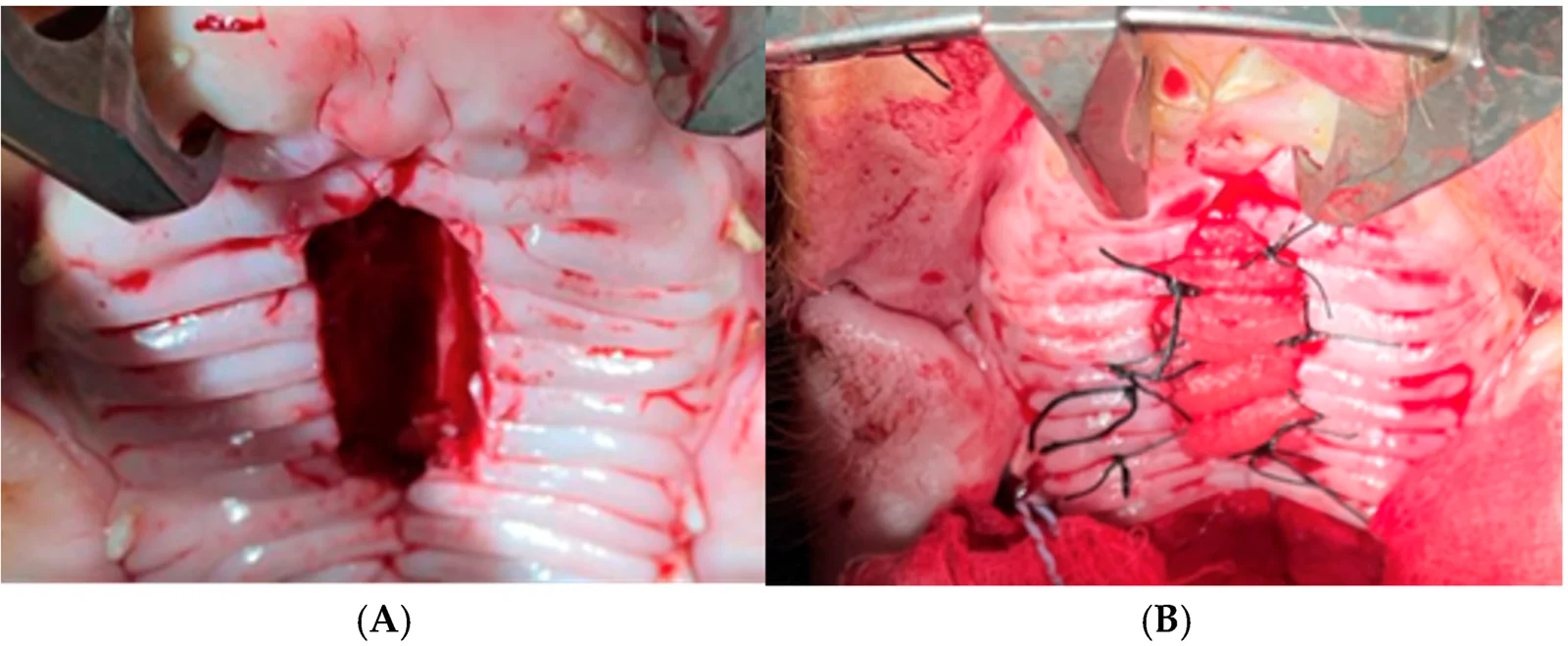
BEMS transplantation procedure: (**A**) iatrogenic hard-palate cleft; (**B**) BEMS positioned and sutured into the palatal defect.

This surgical time required that 3 procedures for the recipients (infant age) follow one another:

(1) Creation of a percutaneous endoscopic gastrostomy (PEG)—All recipient pigs underwent PEG placement before iatrogenic cleft palate induction to ensure nutritional support and prevent bioscaffold infections. The procedures were performed under general anesthesia with orotracheal intubation. Pigs were placed in a supine position, and the abdominal area was marked preoperatively to avoid injury to ribs and liver. The pull technique by Gauderer et al. 1980 was employed [12] using a standard 9.8 mm Olympus endoscope. Procedures were performed by two operators, respectively, managing the endoscope and the percutaneous procedure. The abdomen was disinfected with iopovidone solution, covered with sterile drapes, and a single dose of cefazolin was administered. The stomach was distended with air until it apposed the abdominal wall, with the room lights dimmed to visualize the endoscope’s light through the abdominal wall. After identification of the correct gastrostomy site by pushing the abdominal wall and endoscopically seeing the finger on the anterior wall of the stomach, a 14-gauge trocar was inserted under endoscopic guidance. A guidewire was then inserted through the trocar, retrieved endoscopically, and attached to a 22-French gastrostomy tube. The gastrostomy tube was then rescued by pulling from the mouth to the abdominal wall and fixed to the wall with a dedicated system. To avoid accidental removal of the gastrostomy tube and ensure accurate management of the pigs, the gastrostomy tube was additionally sutured to a skin fold. For the first 24 h, clear liquids were administered, followed by a smooth-textured diet.

(2) Creation of an iatrogenic hard-palate cleft—An iatrogenic cleft of the hard palate was created by surgically removing the mucoperiosteum and palatine bone, measuring 2 cm in length and 1.5 cm in width, establishing persistent communication between the oral and nasal cavities (Figure 1A). The dimensions were chosen to prevent spontaneous healing and to ensure that bone margins were not too far apart, thereby mimicking a wide human cleft palate.

(3) Transplantation of BEMS—After 14 days of culture and MSC seeding, the engineered mucoperiosteal bioscaffold was transplanted into the iatrogenic cleft palate of pigs.

Surgical procedures were performed under general anesthesia, and perioperative as well as postoperative analgesia was administered according to veterinary guidelines. The bioscaffold was positioned to reconstruct the cleft and sutured using interrupted Vicryl^®^ stitches (Figure 1B). Procedures were conducted under sterile conditions to minimize the risk of infection.

### 2.7. Controls

Two control pigs underwent the same preparatory surgical procedures as the recipients, including percutaneous endoscopic gastrostomy (PEG) to ensure adequate nutritional support, followed by creation of an iatrogenic cleft of the hard palate. Subsequently, traditional cleft palate repair was performed using the Langenbeck technique [13]. The controls served as procedural and anatomical references to evaluate the outcomes of standard surgical repair without engineered scaffolds. They were maintained under identical housing and care conditions as the experimental group and observed for the same one-month post-operative period, allowing for direct comparison of tissue healing, bone regeneration, and structural stability.

### 2.8. Histology Live Animal Maintenance and Sacrifice

Postoperative evaluations were conducted at one week, two weeks, and one month for macroscopic findings.

At one month, pigs were euthanized and a Le Fort I osteotomy was performed to remove the reconstructed palates (both by transplantation and by conventional surgery) for macroscopic and microscopic assays.

Histological analysis focused on the description of the epithelial, subepithelial, periosteal, and bone component of the reconstructed palates and on the comparison with the adjacent normal palate.

Histological evaluation was performed by two pathologists who were blinded to treatment allocation. The slides were identified only by animal codes, without information regarding group allocation. Histological assessment was descriptive and qualitative, and no validated histological scoring system was applied.

Angiogenesis, cellular infiltration, and extracellular matrix (ECM) composition were evaluated to characterize the regenerative process. Briefly, palate tissues were placed for 24 h in 10% buffered formalin, treated overnight with ascending alcohol scale, with a clearing agent, then impregnated and embedded in paraffin. Histology and immunohistochemistry-stained slides were acquired on the NanoZoomer S60 (Hamamatsu, Shizuoka, Japan) digital slide scanner platform, equipped with an Olympus 20x/0.75 PlanSAPO objective, a fluorescence imaging module equipped with a L11600 mercury lamp (Hamamatsu), and a linear ORCA-Flash 4.0 digital CMOS camera (Hamamatsu). Immunofluorescence was performed on 2.5 µm-thick sections obtained from formalin-fixed tissue embedded in paraffin. After dewaxing and rehydrating, heat-induced epitope retrieval was performed by boiling the slides with EDTA (pH 9) (Dako, Glostrup, Denmark). Tissue slides were blocked in 5% BSA for 1 h.

### 2.9. Immunofluorescence Assays

Rehydrated coronal sections were blocked at room temperature (RT; i.e., 22–24 °C) for 1 h (1% BSA, 10% goat serum, 0.5% Triton X-100 in PBS) and incubated overnight at 4 °C with primary antibody collagen IV (COLL IV; 1:300, Invitrogen, Waltham, MA, USA for PA5-104508). After washing, sections were incubated with secondary antibody AlexaFluor-546 donkey anti-rabbit IgG (1:500; Invitrogen). DAPI was used for nuclear staining. Images of the sections were captured using a Zeiss Axiophot Microscope (Zeiss, Oberkochen, Germany) with a 5×, 10× and 20× objectives. Subsequently, to obtain images of the entire section, each 10× image of one slice was stitched using Image Composite Editor software (Version 2.0.3.0).

### 2.10. RNA Analyses

#### 2.10.1. RNA Extraction from Bone Tissue

Total RNA was extracted from bone tissue using TRIzol^®^ reagent (Fisher molecular biology) following a phenol–chloroform-based protocol. Briefly, bone samples were snap-frozen in liquid nitrogen and subsequently mechanically disrupted using a pre-chilled mortar and pestle. The powdered tissue was homogenized in TRIzol (1 mL per 100 mg of tissue) using an Ultra-Turrax homogenizer. After centrifugation (1–2 min, 2500 rpm, room temperature), the supernatant was transferred to a new tube and mixed with 200 µL of chloroform. Samples were vigorously shaken, incubated for 5–10 min at room temperature, and centrifuged for 15 min at 12,000 rpm at 4 °C. The aqueous phase was collected, and RNA was precipitated by addition of 500 µL of isopropanol, followed by incubation for 10 min at room temperature. Samples were centrifuged for 15 min at 12,000 rpm at 4 °C. The RNA pellet was washed with 75% ethanol, centrifuged for 5 min at 7500 rpm at 4 °C, air-dried, and resuspended in 30 µL of RNase-free water. RNA was incubated at 65 °C for 5 min to ensure complete solubilization. RNA concentration and purity were assessed spectrophotometrically.

RNA quantity and quality were evaluated using a NanoDrop Lite Plus UV–Vis spectrophotometer (Thermo Fisher Scientific) based on 260/230 and 260/280 absorbance ratios.

#### 2.10.2. Reverse Transcription and RT-qPCR

Equal amounts of RNA (2 μg) were subsequently reverse-transcribed using a high-capacity cDNA reverse transcription kit (Applied Biosystems (ABI), Foster City, CA, USA). Quantitative RT-PCR was performed in triplicates using inventoried TaqMan Gene expression assays purchased fromABI: TBP (Hs00427620_m1) and SPARC (Secreted protein acidic and rich in cysteine; Hs00234160_m1). Relative mRNA levels for genes of interest were normalized to TATA-box-binding protein (TBP) taken as housekeeping genes and calculated by using the 2^−ΔΔCt^ method. Three independent experiments were performed with the ABI 7500 Sequence Detection System Analyzer for RT-qPCR (ABI).

### 2.11. Statistical Analysis

Given the exploratory nature of this pilot study and the limited number of independent biological samples, no formal inferential statistical analysis was performed. Data were evaluated descriptively. Normality testing was not performed because the small group sizes did not allow for a reliable assessment of distributional assumptions. Technical replicates from molecular assays were not considered independent biological units.

## 3. Results

The aim of this work was to assess the in vivo feasibility and early regenerative response of bioengineered natural ECM scaffolds to be used as biomaterials for differentiated stem cells in innovative repair protocols for CL/P. To achieve this goal, eight pigs were recruited: two donors, four recipients, and two controls. In the clinical projection, the donor pigs were adult to simulate adult human donors; the recipient pigs were chosen at 4–6 months of age to simulate infants affected by CL/P.

The mucoperiosteum harvested from the hard palate of donor pigs underwent a standardized protocol for decellularization and was then subjected to recellularization using bone marrow-derived mesenchymal stem cells (MSCs) obtained from the recipient pigs themselves. So, we had a transplantable bioengineered mucoperiosteal scaffold (BEMS). The hypothesis was that BEMS had an osteogenetic potential derived from the presence of periosteum (which physiologically activates during fractures to promote natural bone repair) associated with the presence of stem cells in a stemness environment. The isolated MSCs were differentiated along the osteogenic and adipogenic lineages.

Recipient pigs, subjected to surgical procedure to induce an iatrogenic cleft palate, served as recipients for the engineered mucoperiosteum transplantation. Two pigs, of the same age of the recipients, were used as controls and underwent iatrogenic cleft palate and traditional cleft surgery for palate reconstruction.

Postoperative evaluations were conducted at one week, two weeks, and one month for macroscopic findings.

All animals completed the planned follow-up, and no clinical signs of graft rejection nor overt acute inflammatory reaction were observed on routine histology during the 1-month follow-up.

**Figure 2:**
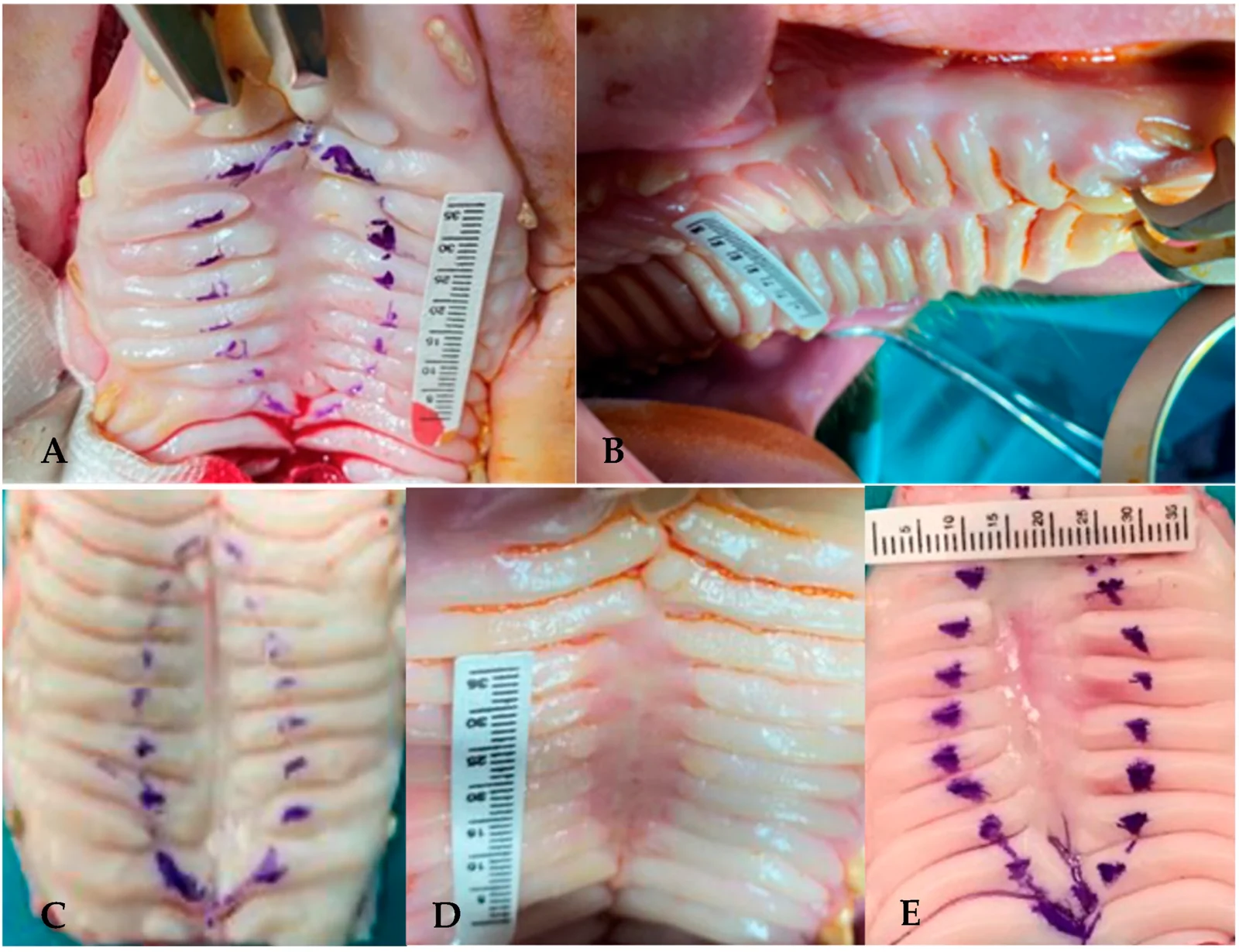
One-month follow-up: Macroscopic view showing well-demarcated transplantation borders (outlined in blue), with complete re-mucosalization and re-formation of palatal rugae (**A**); lateral view demonstrating restoration of tissue height and thickness at the transplaneted site (**B**); Case 2 (**C**); Case 3 (**D**); Case 4 (**E**).

**Figure 3:**
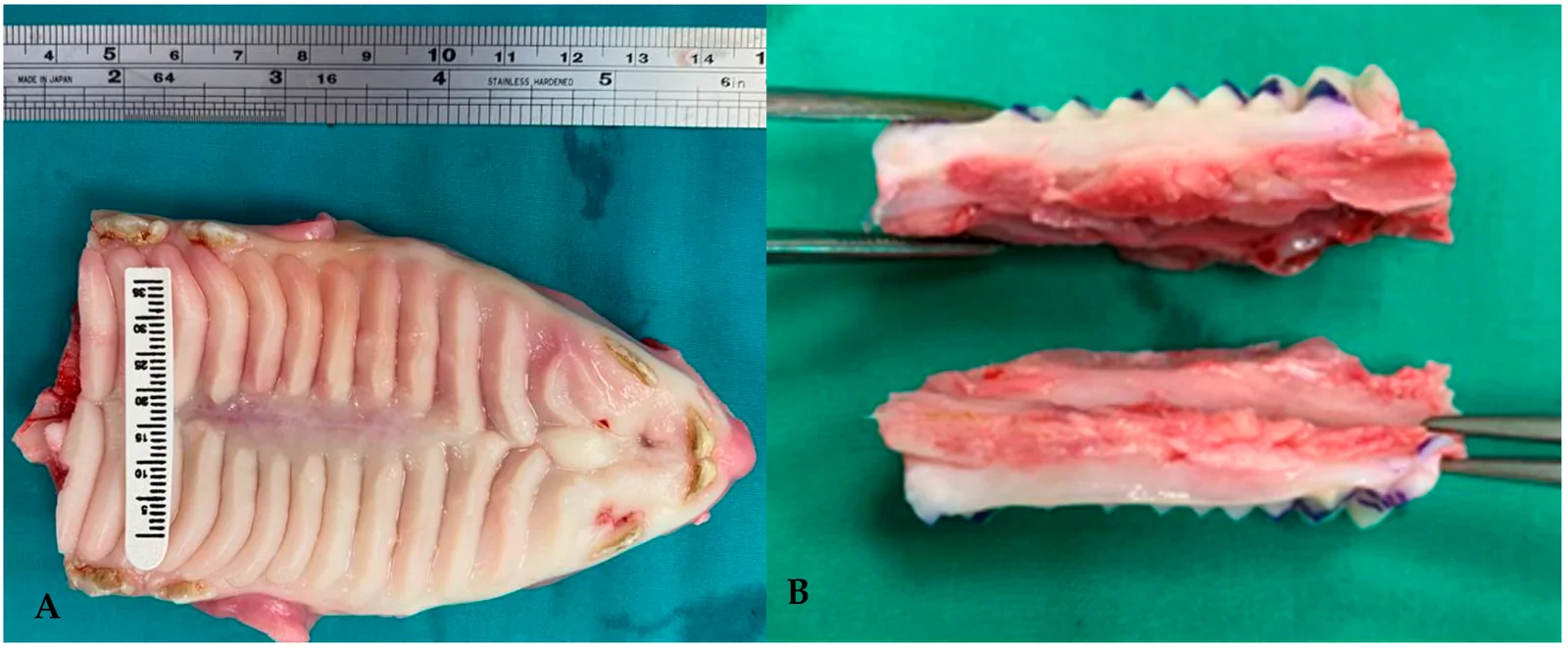
Macroscopic assessment of the reconstructed palate: (**A**) Le Fort I osteotomy showing an intact reconstructed bone segment; (**B**) longitudinal section showing continuity between transplanted and native tissues.

Macroscopic findings in transplanted pigs at 7 and 15 days showed good integration with surrounding tissues. At 1 month, full-thickness tissue regeneration was observed, with evidence of re-mucosalization (Figure 2), as well as bone solidity and stability at the Le Fort I osteotomy site. Specifically, the regenerated palates, harvested through Le Fort I osteotomy, were stable samples resistant to fractures (Figure 3A). From the sagittal section, significant scarring in height was observed with the absence of depressions (Figure 3B).

**Figure 4:**
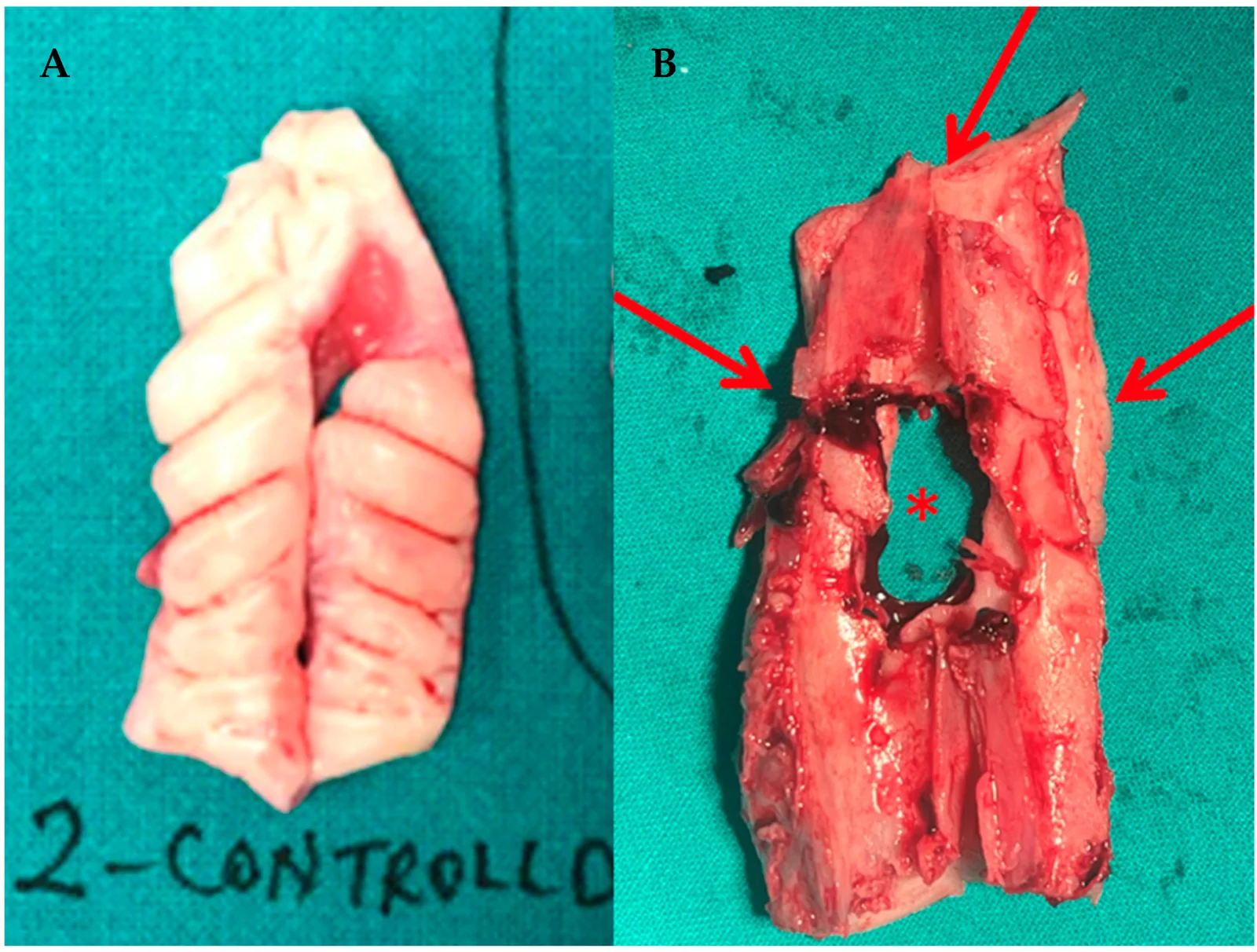
Control cases showing (**A**) an oronasal fistula and (**B**) compromised structural stability after Le Fort I osteotomy, with multiple fracture lines (arrows) and persistence of the osseous gap (*).

In control pigs with standard mucosal reconstruction, we observed oronasal fistula formation and multifragmentary fracture at the Le Fort I osteotomy in all cases (Figure 4).

At one month, the reconstructed palates (both by transplantation and by conventional surgery) were removed for macroscopic and microscopic findings. Histological analysis focused on the description of the epithelial, subepithelial, periosteal, and bone component of the reconstructed palates and on the comparison with the adjacent normal palate. Additionally, angiogenesis, cellular infiltration, and extracellular matrix (ECM) composition were evaluated to better characterize the regenerative process.

Ultrastructural analysis, based on immunofluorescence and molecular analysis, and cCOLL IV immunofluorescence were used to assess basement membrane organization and extracellular matrix remodelling.

**Figure 5:**
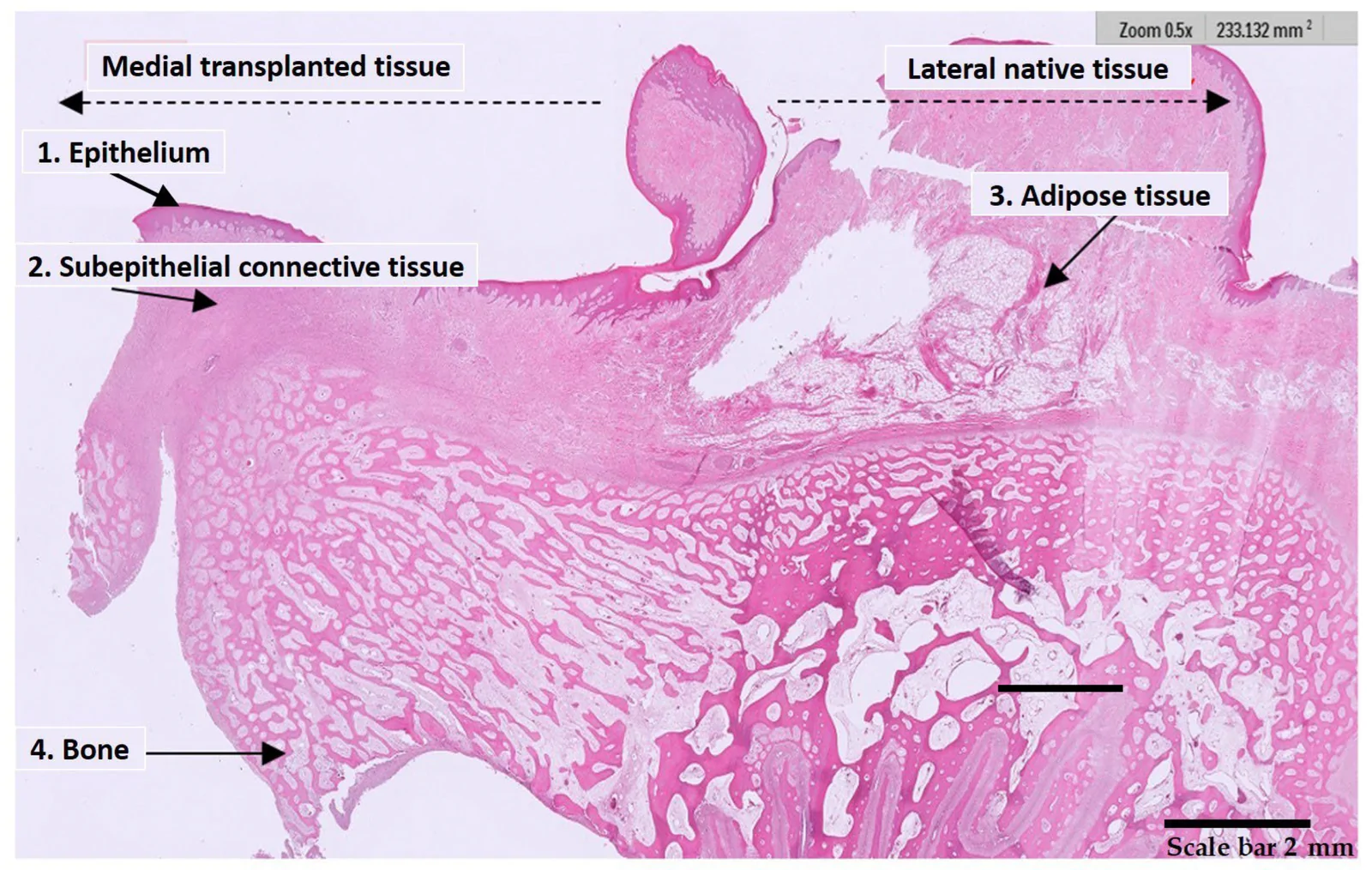
Comparative histology of the medial transplanted and lateral native palate, showing the epithelial layer (1), subepithelial connective tissue (2), adipose tissue (3), and bone (4). Scale bar = 2 mm.

Histological examination of palatal samples revealed tissue organization and remodelling patterns in the transplanted pigs (Figure 5).

- Histological findings

**Figure 6:**
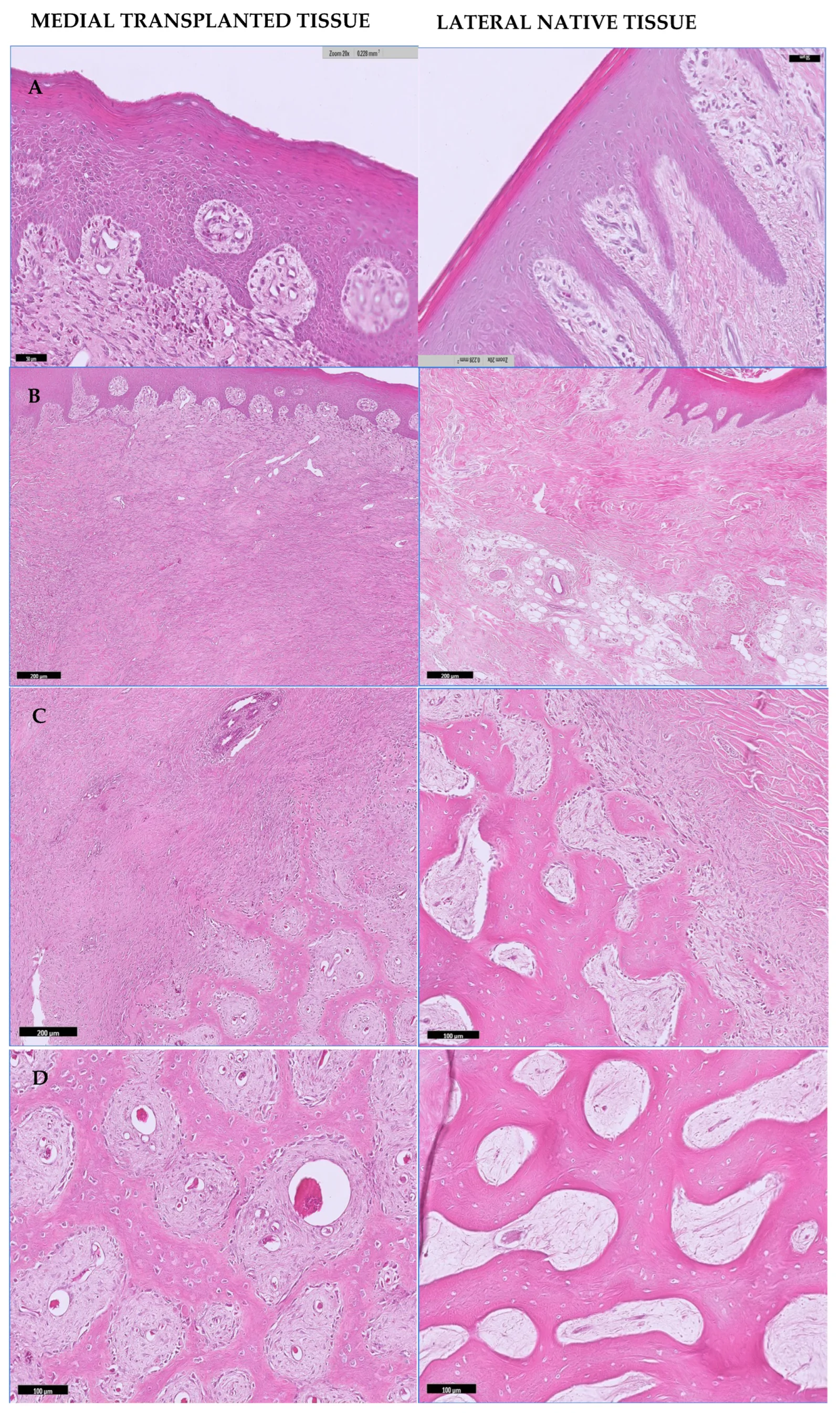
Histological comparison between medial transplanted tissue (**left**) and lateral native palate (**right**). (**A**) Epithelium: the transplanted region shows mild epithelial hyperplasia and focal parakeratosis compared with the more regularly organized native epithelium. (**B**) Subepithelial connective tissue: increased collagenous tissue density and cellularity, with reduced adipose tissue, are observed in the transplanted region. (**C**) Periosteal interface: the periosteum is attenuated and less clearly demarcated in the transplanted area, merging with the overlying fibrotic connective tissue, whereas the native region shows a more distinct connective tissue–periosteum–bone transition. (**D**) Bone: the transplanted region shows thin trabeculae of primary woven bone with osteoblastic rimming, whereas the native palate displays a more mature lamellar/trabecular architecture. Scale bars are shown in each panel.

Epithelium: In the transplanted group, the regenerated epithelium exhibited mild hyperplasia and parakeratosis, with a well-organized basal layer and progressive maturation towards the superficial layers in all cases (Figure 6A). In the control group, the epithelial layer displayed discontinuous areas of delayed re-epithelialization correlated with the presence of oronasal fistulas in Case 1, and irregular areas of epithelial atrophy on the median line (in correspondence of the sutures) in Case 2.

Subepithelial Connective Tissue: In transplanted pigs, the connective tissue was characterized by dense collagenous fibrosis with plump fibroblasts and loss of the adipose tissue in comparison with the adjacent normal mucosa, and minimal chronic inflammation in some cases. The collagen fibres appeared well-organized and densely packed, supporting epithelial stabilization and mucosal integrity (Figure 6B). Meanwhile, the controls showed looser connective tissue architecture, with persistent inflammatory infiltrate (mainly lymphocytes and macrophages), and delayed remodelling.

Angiogenesis and Vascularization: In the transplanted group, CD31 immunostaining revealed a capillary network within the neotissue. Neovascularization was also observed in the scar tissue in control pigs.

Periosteum: The periosteal layer in transplanted pigs appeared attenuated or absent, merging with the fibrosis of the subepithelial connective tissue (Figure 6C). In the controls, the periosteum was discontinuous in the median line.

Bone: In the transplanted group, histological examination revealed thin trabeculae of primary woven bone, which were continuous with adjacent lamellar bone. The osteoblastic rim was well evident, and osteocytes exhibited an active, plump morphology, indicative of ongoing bone formation. Mild fibrosis was observed in the intertrabecular spaces (Figure 6D).

In contrast, controls pigs exhibited persistence of thin fibrous tissue and a total lack of structured bone or ossification signs.

- Immunofluorescence findings

**Figure 7:**
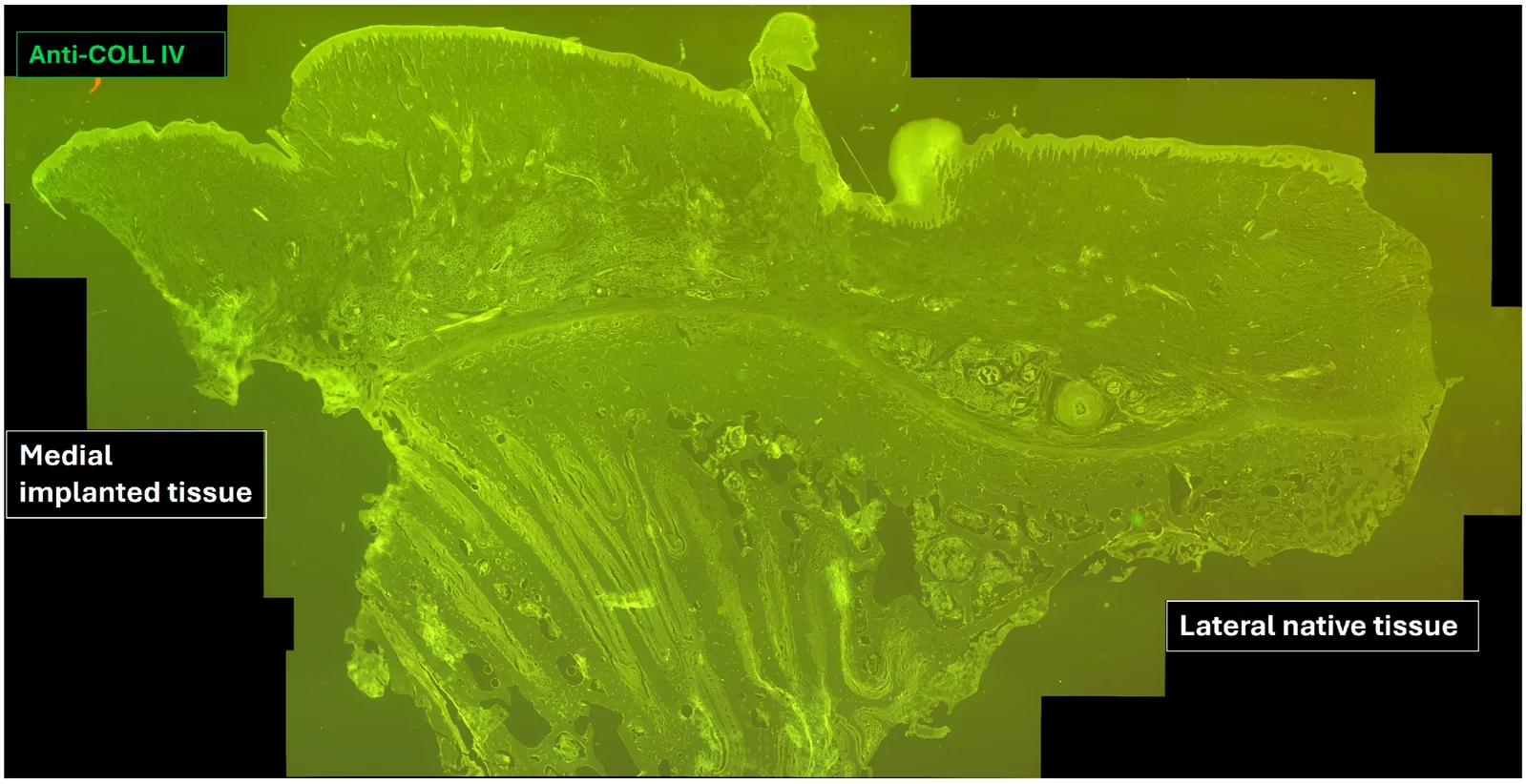
Representative immunofluorescence image showing the stitching COLL IV staining in the medial transplanted region and lateral native tissue. Original magnification, 5×.

**Figure 8:**
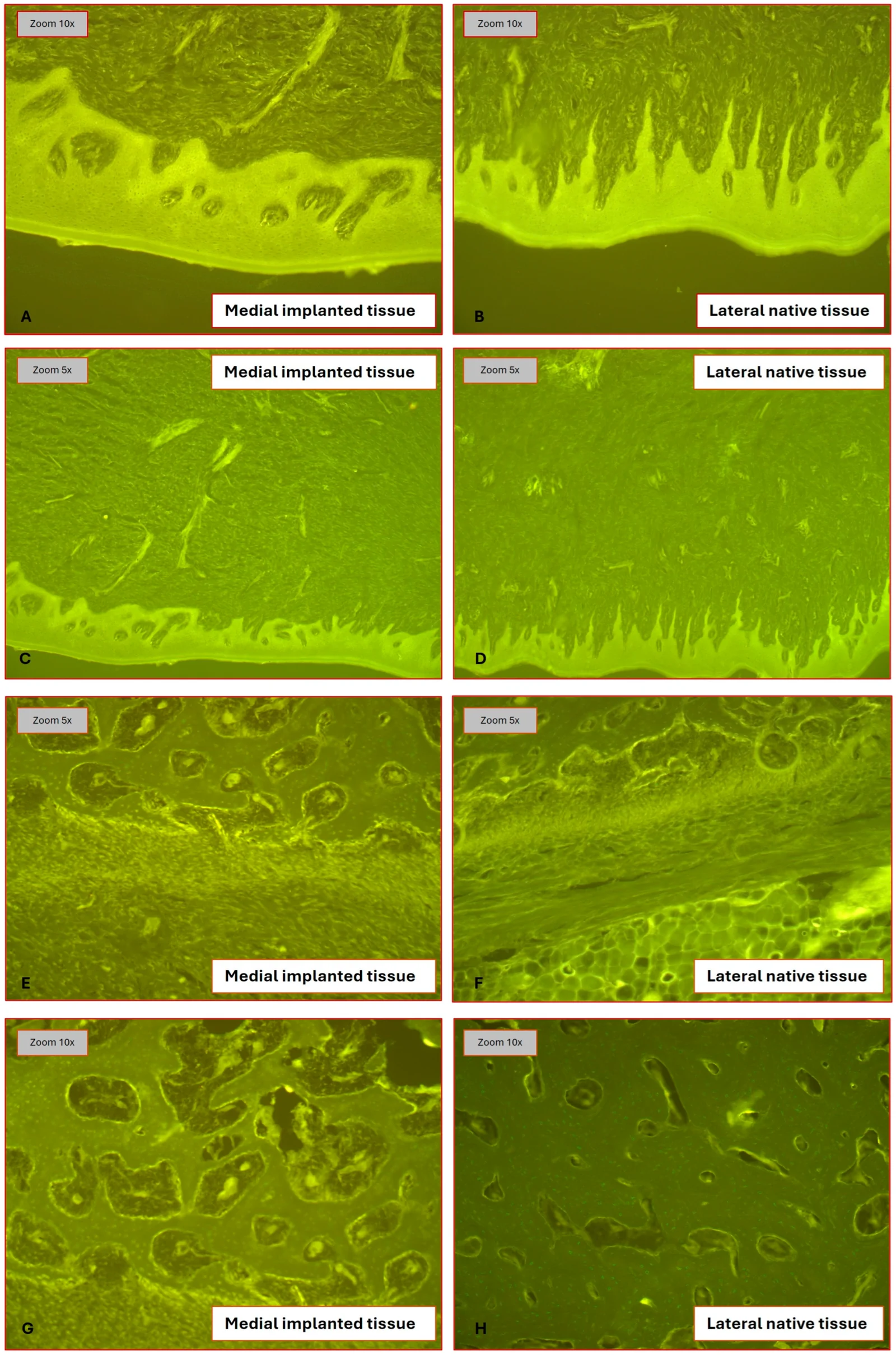
Representative COLL IV immunofluorescence images comparing medial implanted tissue and lateral native tissue. (**A**,**B**) Epithelial layer, original magnification 10×; (**C**,**D**) subepithelial connective tissue, original magnification 5×; (**E**,**F**) periosteal region, original magnification 5×; and (**G**,**H**) bone tissue, original magnification 10×.

**Figure 9:**
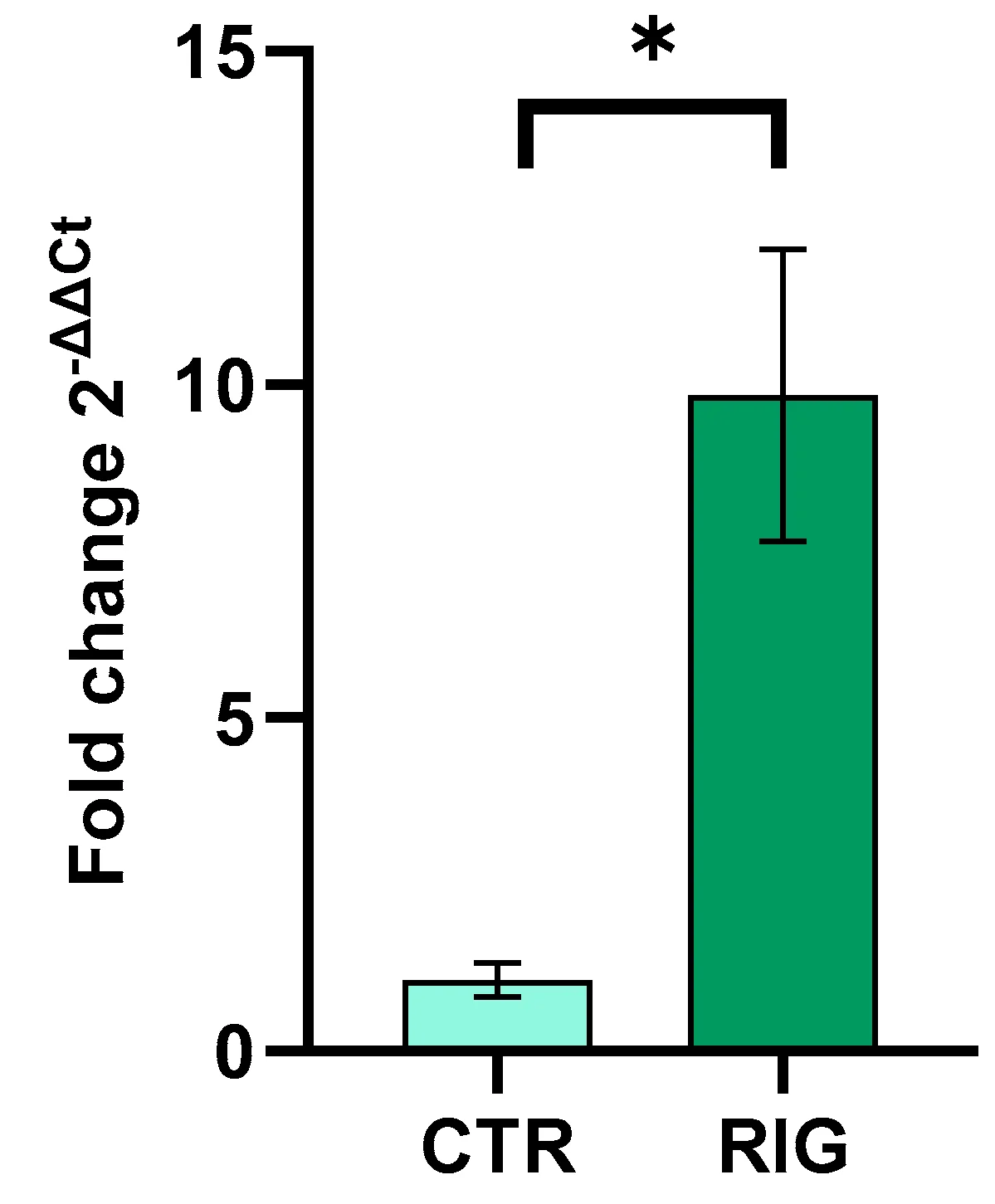
Relative SPARC mRNA expression in regenerated tissue (RIG) compared with control tissue (CTR), expressed as fold change using the 2^−ΔΔCt^ method. Data are presented as mean ± SEM. * *p* = 0.0165, Student’s *t*-test.

Immunofluorescence analysis of the engineered tissues was performed using an antibody for collagen IV, a marker associated with basement membrane organization and tissue remodelling. These findings provided further evidence supporting palatal regeneration and were consistent with the histological observation (Figure 7 and Figure 8). Moreover, molecular analyses showed increased SPARC expression in transplanted tissues, suggesting ongoing osteogenic differentiation and bone remodelling processes (Figure 9).

## 4. Discussion

Conventional cleft surgery does not restore all anatomical planes of the hard palate to full thickness. Specifically, none of the surgical procedures used for cleft palate repair lead to definitive correction of hard palate bone interruption. So, the closure of the cleft consists of repairing the available soft tissues under tension.

In the standard surgery for CL/P, two flaps of palatal mucoperiosteum are brought together in the technique most commonly performed worldwide, and the bone gap remains for life, with discomfort.

We study how regenerative medicine and tissue engineering can help us achieve a reconstruction of all the missing tissues, which is currently not possible with traditional surgery.

Challenges of Current Surgical Approaches: Cleft palate repair remains one of the most complex reconstructive challenges in craniofacial surgery. Conventional surgical techniques, including the Von Langenbeck, Bardach, Veau–Wardill–Kilner, and Furlow double-opposing Z-plastic procedures, primarily aim to close the palatal defect using local soft-tissue flaps [14]. However, none of these techniques successfully restore bony continuity in the hard palate, leading to the possibility of persistent oronasal fistulas (ONFs), maxillary hypoplasia, and impaired speech development [15].

One of the most common complications following cleft repair is ONF formation, which can be related to a mechanism of repair under tension and lack of available tissue due to wide clefts, with reported incidence rates ranging from 0% to 78% and the reported overall success rate of ONF repair approaching 85%, with a recurrence rate of 33% to 37%, so the treatment of ONF remains a challenging problem [16,17,18,19,20,21,22].

Moreover, palatal wound contraction during healing can impair maxillary bone growth, leading to dental arch malalignment and resulting in midfacial retrusion [23].

In recent years, advancements and alternative regenerative techniques have been proposed for cleft palate repair, exclusively for alveolar cleft reconstruction. These include autologous bone grafting, primarily using iliac crest or rib bone; synthetic bone substitutes such as hydroxyapatite, β-tricalcium phosphate, bioactive silicates, polymers (PGA and PLA), bioglass scaffolds, or biocomposites; platelet-rich fibrin (PRF) scaffolds; and decellularized extracellular matrices (dECMs)—all of which have resulted in suboptimal regenerative outcomes [24,25,26,27,28,29,30,31,32]. Importantly, these approaches are limited to alveolar clefts and do not address the hard palate. Nothing effectively restores the palatine bony laminae, except for the historic tibial periosteal graft [33], which provided only a thin continuity and has since been abandoned due to the need for partial periosteal harvesting from the newborn’s tibia, which over time resulted in a slight shortening of tibial length [33,34].

Advances in biomaterial-based scaffolds have attempted to overcome the previous limitations; various synthetic biomaterials, such as hydroxyapatite, β-tricalcium phosphate, and bioactive glasses, have demonstrated osteoconductive properties but often lack sufficient mechanical strength and biointegration capacity [35]. Recent reviews on structural scaffold biomaterials for bone defect repair stress that scaffold design should couple mechanical/structural performance (including compression, fatigue, and permeability-related behaviour) with biological function, with particular attention to osteogenic capacity [36]. Recently, a lyophilized platelet-rich fibrin (LyPRF) scaffold has also been proposed for craniofacial regeneration, showing promising results in supporting osteogenesis and tissue repair [37]. However, challenges such as inadequate vascularization and immunogenic responses persist with these synthetic and composite biomaterials [35]. Along these lines, periosteum-inspired approaches have been proposed that aim to trigger early angiogenesis to establish a vascularized fibrous layer and then support the in vivo formation of a cambium-like layer by endogenous mesenchymal cells; one example is a hierarchical micro/nanofibrous membrane enabling sustained VEGF release [38]. In an orthotopic xenograft platform for human tissue-engineered periosteal constructs, a multiphasic design combining human endothelial cells (to model the vascular niche) with human BM-MSCs (to model the osteogenic niche) showed that BM-MSCs retained a precursor phenotype in vivo, while endothelial cells formed mature vessels that connected with host vasculature; moreover, adding an in vitro-engineered endothelial network increased vascularization compared with cell-free constructs [39].

To develop new methodological approaches in this complex scenario, our studies build upon the principle that decellularized palatal mucoperiosteal scaffolds, engineered by microperforation treatment in vitro [9], not only support osteogenic differentiation but also show a low-immunostimulatory profile, which may represent an advantage over previous tissue-engineered constructs. In fact, this decellularized bioscaffold is able to preserve the structural, biochemical, and biomechanical properties of the native tissue, therefore providing a suitable environment for subsequent MSC repopulation by osteogenic differentiation. In our previous in vitro characterization, we observed the lack of hematopoietic and immunostimulatory markers, including CD34, CD45, CD80, CD86, and HLA-DR, supporting a low-immunostimulatory profile of the MSC-seeded scaffold platform before in vivo implantation.

Our decellularization protocol effectively removed native cells and genetic material from the extracellular matrix (ECM), resulting in a natural, decellularized scaffold. Previous studies have established a threshold of 50 ng of double-stranded DNA (dsDNA) per mg of ECM dry weight as a key criterion to assess the adequacy of decellularization and to minimize the risk of adverse immune responses [40]. This represents a significant advancement over earlier tissue-engineered scaffolds, which often failed due to immune rejection and suboptimal host integration.

In our previous validation study, molecular analysis of the decellularized palatal scaffolds revealed only trace amounts of residual donor DNA, consistently remaining below the critical 50 ng/mg threshold. These findings confirm the effectiveness of our decellularization protocol for palate-derived tissues. Furthermore, histological examination following QMR^®^ microperforation demonstrated no evidence of necrosis or thermal damage, suggesting that the ECM maintained its structural integrity and could be well tolerated by the host in potential clinical applications. The application of QMR^®^ technology enhanced both the porosity and osteoinductive potential of the bioscaffold. This microperforation technique facilitated deeper infiltration of mesenchymal stem cells (MSCs) and improved scaffold vascularization, thereby addressing a major limitation of traditional scaffold-based approaches [9]. More generally, across tissue-engineering applications, scaffold architecture and physical/topographical cues are discussed as key determinants of cell adhesion, growth and differentiation [41].

Subsequently, the palate scaffolds were subjected to a recellularization protocol, which involved seeding the decellularized scaffolds with MSCs previously isolated from the bone marrow (BM). The use of these cells offers several advantages, including their ability to orchestrate inflammatory processes that regulate all phases of tissue regeneration, their high resistance to neoplastic transformation following in vitro exposure to physical and chemical stress, and their capacity to enhance immune tolerance to non-self-antigens [42,43,44,45]. Moreover, the differentiation potential of BM-MSCs is particularly relevant in supporting the osteogenic and chondrogenic components of the tissues targeted for regeneration [46,47].

The hBM-MSCs used in our previous study were successfully isolated and expanded ex vivo through several passages. Before seeding, these cells were characterized and tested for their ability to differentiate into adipocytes and osteocytes. The recellularization of palate scaffolds was evaluated, and the seeded BM-MSCs demonstrated good biocompatibility and deep tissue penetration. Lastly, microscopic analysis revealed that both medium supplements used for cell culture and seeding (i.e., FBS and hPL) supported MSC growth and adhesion during both the ex vivo expansion and seeding phases. This aspect is of particular relevance, considering that the use of xeno-free products is essential for the future clinical application of these engineered scaffolds. Our decellularized and microperforated bioscaffold showed the presence of a bioactive ECM network, which provided a favourable microenvironment for effective MSC engraftment, as revealed by ultrastructural analysis. Moreover, this mucoperiosteum, engineered through QMR^®^ perforative treatment, exhibited increased osteoinductive potential for mesenchymal precursor cells. Our molecular results showed a significant increase in the expression levels of both COL1A1 and SPARC, especially on day 14 [9].

These osteoblast-related genes are considered master regulators of osteoblast differentiation and early transcription factors for the commitment to the osteoblast lineage [48,49].

Specifically, SPARC overexpression was found to inhibit cell proliferation while inducing osteogenic differentiation of hBM-MSCs [49,50].

We also observed a reduction in the expression of the stemness gene Sox2, indicating the ability of the proposed scaffold to induce cell differentiation even in the absence of additional microenvironmental signals.

Comparable molecular findings were reported by Mattioli-Belmone et al. in a study utilizing a demineralized bone matrix seeded with human umbilical cord-derived MSCs [51].

The seeded bone marrow-derived MSCs (BM-MSCs) exhibited strong adhesion and proliferation within the scaffold microstructure; enhanced osteogenic differentiation, as evidenced by upregulation of SPARC and COL1A1; and downregulation of Sox2, suggesting progression toward terminal osteoblast differentiation [50].

Based on these findings, we hypothesize that in vivo transplantation of BEMs may function as both a recruiter of mesenchymal precursors and a differentiation inducer, enhancing the expression of osteogenic markers and promoting palate regeneration.

Accordingly, we developed an efficient protocol to decellularize and repopulate this bioscaffold with MSCs and investigated its in vivo properties of BEMs by transplantation in young pigs with iatrogenic cleft palate.

The combination of viable MSCs, the preserved ECM architecture, and minimal residual donor DNA supported both mucosal and bone regeneration.

The experience gathered with this technology, following one-month transplantation, revealed favourable macroscopic and histological outcomes, including increased cellular thickness compared to control cases and restoration of all anatomical layers, including the palatine bone.

Histological and ultrastructural analyses confirmed complete mucosal regeneration and indicated that the engineered scaffolds promoted organized primary bone formation, characterized by trabecular networks and active osteoblast recruitment.

The upregulation of SPARC—a critical regulator of osteogenesis—suggests a strong bioactive interaction between the scaffold and host cells. Importantly, the absence of early signs of immune rejection provides preliminary evidence of short-term in vivo tolerance, supporting the scaffold as a promising candidate for further preclinical investigation and potential future clinical translation; however, specific immunological analyses are required for definitive assessment.

Furthermore, our study highlights the essential role of angiogenesis in the regenerative process. Immunohistochemical analysis demonstrated enhanced vascularization, confirmed by CD31 staining within the transplanted tissues—an essential feature for long-term graft survival and functionality.

These findings suggest that BEMs may serve as an adjunct to conventional cleft palate repair, reducing the need for secondary surgeries, minimizing donor site morbidity, and improving long-term functional outcomes.

### Limitations and Challenges

While the results of our study are promising and provide preliminary evidence supporting the feasibility of engineered mucoperiosteal scaffolds for full-thickness cleft palate regeneration, several limitations and challenges must be acknowledged to ensure a rigorous and balanced interpretation of the data.

Given the pilot nature of the study and the limited sample size (four treated animals and two controls), these findings should be interpreted as preliminary evidence of feasibility rather than definitive evidence of efficacy. The study was not powered to provide robust between-group statistical comparisons, and larger controlled studies with longer follow-up are required to confirm the regenerative potential and translational relevance of this approach. In particular, the findings regarding osteogenic differentiation should be considered preliminary and require confirmation using a broader panel of osteogenic markers. Molecular characterization was limited to the available RT-qPCR analysis, without a broader RNA-expression panel or protein-level confirmation by Western blot.

Although no clinically detectable adverse reactions were observed, specific immunological analyses were not performed; therefore, the present findings should be interpreted as preliminary evidence of in vivo tolerance rather than definitive evidence of non-immunogenicity.

A further limitation is the absence of micro-CT or other radiological assessment of bone regeneration. Although histological analysis provided evidence of newly formed bone tissue, the lack of three-dimensional quantitative imaging prevented a comprehensive assessment of bone volume, architecture, and mineralization. Future studies should incorporate micro-CT analysis to confirm and quantitatively characterize these preliminary findings.

An additional limitation is the relatively short follow-up period of one month. The one-month post-implantation follow-up provided insight into early healing and scaffold integration and allowed histological identification of an early osteogenic response; however, it did not allow for assessment of long-term bone maturation, remodelling, resorption dynamics, or functional stability. Longer-term studies will therefore be required to determine the stability and maturation of the newly formed bone and to evaluate the durability of the regenerated tissue, its biomechanical properties, and the capacity of the scaffold to support ongoing craniofacial development, particularly in a growing organism.

Vascularization and host–graft integration represent another major challenge. Although CD31 immunostaining qualitatively identified neovascularization within the transplanted tissues, no quantitative assessment of vascular density or angiogenesis was performed. Therefore, it remains unclear whether the observed neovascularization is sufficient and stable in the long term, especially in larger or more complex defects. Strategies to improve vascular supply—such as revascularized constructs, incorporation of angiogenic growth factors (e.g., VEGF, BMP-2), or co-seeding with endothelial progenitor cells—should be explored to optimize tissue perfusion, enhance MSC survival, and support sustained bone formation.

The choice of the porcine model, while advantageous due to its anatomical and physiological similarities to humans, still presents limitations. Pigs do not perfectly replicate the complexity of human cleft palate healing, particularly in terms of genetic variability, immune response, and speech-related outcomes. Thus, translating our findings to the clinical setting will require additional testing in patient-derived ex vivo systems, advanced humanized animal models, and ultimately, early-phase clinical trials to confirm safety, efficacy, and functional recovery.

Scaffold standardization and reproducibility remain technical aspects to be refined. Ensuring consistent decellularization, uniform microperforation, and homogenous MSC seeding across multiple samples is essential to support the scalability of this approach. Additionally, the identification of quality control parameters for scaffold production, including mechanical integrity, DNA quantification, and residual detergent levels, will be necessary for regulatory approval and clinical translation.

Finally, ethical and logistical considerations related to the sourcing of donor tissues, large-scale MSC expansion, and the regulatory framework for xenogeneic-derived scaffolds should be addressed. This will require collaboration with transplant centres to source human donor tissue in translation.

Autologous MSC therapy represents a compelling solution for immune compatibility but may present challenges in terms of cost, time, and accessibility.

Taken together, the limitations of this pilot study should be considered in the context of its exploratory and feasibility-oriented design. Despite the limited sample size, short follow-up, and absence of some quantitative assessments, the findings provide preliminary evidence supporting the feasibility of the BEMS approach and establish a basis for future studies with larger cohorts, longer follow-up, and comprehensive quantitative, histomorphometric, vascular, molecular, and immunological analyses.

Overall, while this study marks a significant advancement in the field of craniofacial regenerative medicine, a multidisciplinary effort combining bioengineering, surgery, immunology, and clinical science will be essential to overcome current barriers and move toward routine clinical application.

To further validate our findings, long-term studies will be conducted to assess the implant–tissue interactions and the hierarchical structure of the regenerated bone. These investigations aim to establish a clinically viable strategy for treating hard palate defects by optimizing the biophysical and biological properties of the engineered scaffold.

This study’s outcomes are significant not only from a scientific perspective advancing regenerative medicine and tissue engineering but also from a socio-medical standpoint, offering a novel therapeutic approach for congenital malformations, post-oncological defects, and post-traumatic conditions.

## 5. Conclusions

This study presents a paradigm shift in cleft palate repair, providing preliminary evidence supporting the potential of engineered mucoperiosteal scaffolds seeded with mesenchymal stem cells—BEMS—to achieve full-thickness tissue regeneration. Unlike traditional surgical approaches that primarily focus on soft-tissue closure, this pilot large-animal study provides preliminary evidence that BEMS implantation is feasible and associated with early multilayer palatal repair and woven bone formation at 1 month, providing an osteoinductive solution that overcomes the key limitations of conventional cleft palate surgery.

All transplanted pigs successfully formed primary bone tissue, whereas control pigs exhibited incomplete and structurally compromised regeneration. These findings provide preliminary evidence that BEMS may support both soft-tissue and early bone regeneration; however, confirmation in larger controlled studies is required.

This study provides preliminary preclinical evidence supporting the feasibility and early regenerative potential of BEMS in full-thickness cleft palate repair. By integrating decellularization and QMR^®^-assisted microperforation with mesenchymal stem cell repopulation, we present a novel platform that was well tolerated in vivo and was associated with early multilayer palatal repair, including bone formation.

The innovation lies in the synergistic use of biomaterial engineering and stem cell therapy. These preliminary in vivo findings support further investigation of the translational potential of this platform.

As regenerative medicine continues to evolve, this work lays a foundational framework for future human trials and represents a significant step toward biomaterial-based, patient-specific therapies in craniofacial reconstruction. With further refinement and long-term validation, this technology could redefine the standard of care in cleft palate surgery, providing durable, biologically integrated solutions for congenital and acquired craniofacial defects.

## Data Availability

All data generated or analyzed during this study are included in this published article.

## Abbreviations

The following abbreviations are used in this manuscript:

ABI	Applied Biosystems
BEMS	Bioengineered mucoperiosteal scaffolds
BM	Bone marrow
BM-MSCs	Bone marrow-derived mesenchymal stem cells
BMP-2	Bone morphogenetic protein 2
BSA	Bovine serum albumin
CD31	Cluster of differentiation 31
CD34	Cluster of differentiation 34
CD45	Cluster of differentiation 45
CD80	Cluster of differentiation 80
CD86	Cluster of differentiation 86
cDNA	Complementary DNA
CL/P	Cleft lip and palate
CMOS	Complementary metal-oxide-semiconductor
COL1A1	Collagen type I alpha 1 chain
COLL IV	Collagen IV
DAPI	4′,6-diamidino-2-phenylindole
dECM	Decellularized extracellular matrix
DMEM	Dulbecco’s Modified Eagle Medium
DNA	Deoxyribonucleic acid
DNase-I	Deoxyribonuclease I
dsDNA	Double-stranded DNA
ECM	Extracellular matrix
EDTA	Ethylenediaminetetraacetic acid
FBS	Fetal bovine serum
hBM-MSCs	Human bone marrow-derived mesenchymal stem cells
HLA-DR	Human leukocyte antigen-DR
INN	International Nonproprietary Name
IRCCS	Istituto di Ricovero e Cura a Carattere Scientifico
KU	Kunitz units
LyPRF	Lyophilized platelet-rich fibrin
mRNA	Messenger RNA
MSC	Mesenchymal stem cells
ONF	Oronasal fistula
OPBA	Organismo Preposto al Benessere Animale
P/S	Penicillin–streptomycin
pBM-MSCs	Porcine bone marrow-derived mesenchymal stem cells
PBS	Phosphate-buffered saline
PEG	Percutaneous endoscopic gastrostomy
PGA	Polyglycolic acid
PLA	Polylactic acid
PRF	Platelet-rich fibrin
QMR^®^	Quantum Molecular Resonance
RNA	Ribonucleic acid
RT	Room temperature
RT-PCR	Reverse transcription polymerase chain reaction
RT-qPCR	Reverse transcription quantitative polymerase chain reaction
SPARC	Secreted protein acidic and rich in cysteine
TBP	TATA-box binding protein
UV–Vis	Ultraviolet–visible
VEGF	Vascular endothelial growth factor
